# Relationship of the Median and Radial Nerves at the Elbow: Application to Avoiding Injury During Venipuncture or Other Invasive Procedures of the Cubital Fossa

**DOI:** 10.7759/cureus.1094

**Published:** 2017-03-13

**Authors:** Vlad Voin, Joe Iwanaga, Juan P Sardi, Christian Fisahn, Marios Loukas, Rod J Oskouian, R. Shane Tubbs

**Affiliations:** 1 Anatomical Research, Seattle Science Foundation; 2 Seattle Science Foundation; 3 Neurociencias, Pontificia Universidad Javeriana; 4 Orthopedic Surgery, Swedish Neuroscience Institute; 5 Department of Anatomical Sciences, St. George's University School of Medicine, Grenada, West Indies; 6 Neurosurgery, Complex Spine, Swedish Neuroscience Institute; 7 Neurosurgery, Seattle Science Foundation

**Keywords:** anatomy, complications, avoidance, veins, nerve injury

## Abstract

**Introduction:**

The median and radial nerves are two important neural structures found in the cubital fossa. The trajectory and landmarks used to identify their location are important when procedures are done in this area.

**Methods and materials:**

Ten fresh-frozen cadavers were dissected (20 upper limbs) and measurements were taken from the medial epicondyle to the median and radial nerves as well as to the lateral epicondyle of each limb.

**Results:**

The distance between the medial epicondyle and the median nerve was found to be three centimeters with a range of 2.1 to four centimeters and the distance from the medial epicondyle to the radial nerve had a mean distance of 5.5 cm and a range of 3.8 to seven centimeters.

**Discussion:**

Damage to the median or radial nerves can lead to major complications including loss of extension, flexion, and sensation in the forearm and hand. Other studies have tried to identify the course of these nerves in order to prevent their injury during procedures.

**Conclusion:**

After identifying the medial epicondyle, using the results we obtained, physicians may have a better understanding of where the median and radial nerves lie within the cubital fossa when performing procedures in this area.

## Introduction

According to Hackl, et al., the course of the radial and median nerves through the elbow joint can vary [1]. These two nerves arise from the brachial plexus and supply sensory and motor innervation to the arm, forearm, and hand. The radial nerve, derived from C5-T1 is one of the terminal branches of the posterior cord of the brachial plexus [2]. The median nerve is formed by the union of the lateral and medial roots, which meet anteriorly to the third part of the axillary artery [2]. Both nerves give off branches proximally and distally to the elbow joint.

The median and radial nerves can easily be injured by trauma or iatrogenically. The elbow joint is particularly in a relevant location where these two nerves are at high risk for being injured, either during surgery or venipuncture. Both arthroscopic and open elbow surgeries predispose them to damage. A proper understanding of the anatomy at the elbow can minimize the risk of such complications.

In this anatomical study, we measure the distances of the radial and median nerves at the elbow joint to elucidate their locations and their relationships to other structures at that joint. The goal of this study is to alert clinicians and surgeons to the location of these nerves during certain procedures on the elbow by mapping their distance from easily palpable bony landmarks. Informed consent statement was obtained for this study.

## Materials and methods

**Figure 1:**
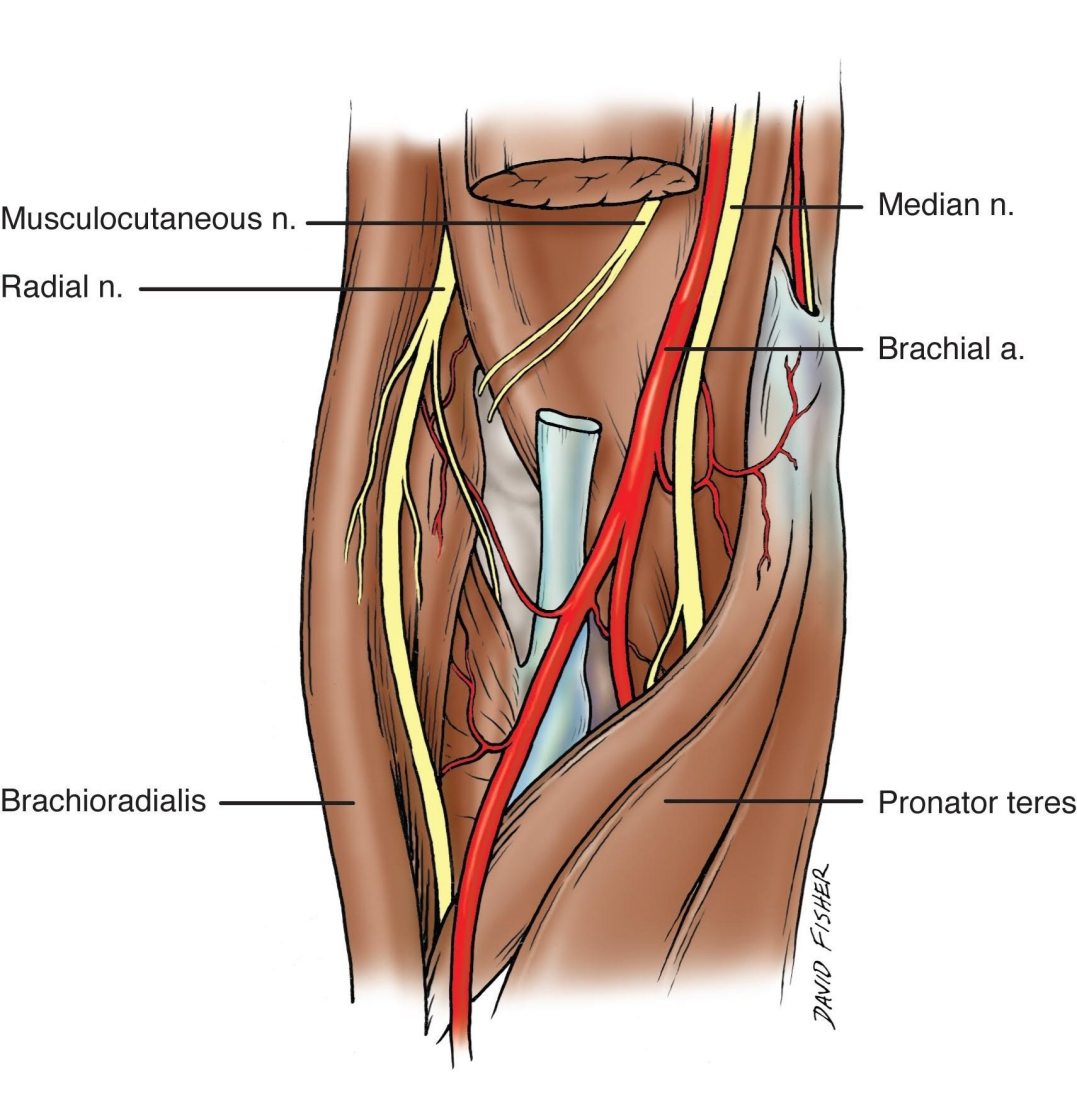
Schematic view of the right cubital fossa. Note that the distal biceps brachii muscle has been removed. In the cubital fossa, the biceps tendon is most lateral and is adjacent to the brachial artery and the more medially placed median nerve. Also note, the radial nerve entering the cubital fossa in a more lateral location compared to the median nerve Original drawing done by David Fisher

Ten fresh-frozen cadavers (20 upper limbs) were dissected for this study. The age range at death was 60-98 years old (mean 79 years). There were five male and five female specimens. With the specimens in the supine position, the cubital fossa was dissected. First, the skin and fasciae were removed and the radial and median nerves identified. Second, markers were placed on the medial epicondyle and measurements made between this bony landmark and these two nerves (Figure. 1). Each measurement was repeated three times and the average was taken. All measurements were made with calipers and a ruler. Statistical analysis between sides and sex were performed with Statistica for Windows with significance set at p <0.05.

## Results

No specimen was found to have pathology or signs of previous surgery to the area dissected. The mean distance between the medial epicondyle and the median nerve was three centimeters with a range of 2.1 to 4.0 cm (table #1). The mean distance from the medial epicondyle to the radial nerve was 5.5 cm and a range of 3.8 to seven centimeters (Table #1). The distance between the medial and lateral epicondyles ranged from six to 10 cm with a mean of 7.7 cm (Table #1). The distance between the medial and lateral epicondyles was statistically greater in males than females, but the distance from the medial epicondyle and median nerve was not statistically significant between the sexes or sides (Table #2, #3). The radial nerve tended to be a greater distance from the medial epicondyle comparing sexes (males > females) and on right sides but neither of these was statistically significant (Table #2). 

**Table 1:** Measurements (in cm) from the medial epicondyle to the median nerve, radial nerve and lateral epicondyle in male and female cadavers. Damaged with dissection

Sex	Arm	Medial Epicondyle to Median Nerve	Medial Epicondyle to radial nerve	Medial epicondyle to lateral epicondyle
Female	Right	3.2	*	*
Female	Left	3.4	*	*
Female	Right	2.7	4.5	6.2
Female	Left	2.7	4.5	6.5
Female	Right	2.9	4.3	6.5
Female	Left	*	*	*
Female	Right	2.7	3.8	6.4
Female	Left	2.5	3.9	6
Female	Right	2.5	4.9	7.9
Female	Left	2.7	4.9	6.8
Male	Right	3.7	6.5	9.2
Male	Left	3.7	6.6	9.5
Male	Right	4	7	10
Male	Left	3.8	6.1	7.8
Male	Right	3	6.3	7.6
Male	Left	2.6	5.7	7.6
Male	Right	2.7	5.7	7.5
Male	Left	2.1	5.3	7.6
Male	Right	2.9	6.3	8.9
Male	Lef	3.9	7	9
Mean Distance		3.0	5.5	7.7

**Table 2:** Mean distance (cm) between males and females

Sex	Medial epicondyle to median nerve	Medial epicondyle to radial nerve	Medial epicondyle to lateral epicondyle
Male	3.2	6.3	8.5
Female	2.8	4.4	6.6

**Table 3:** Mean distance (cm) between right and left arms

Arm	Medial epicondyle to median nerve	Medial epicondyle to radial nerve	Medial epicondyle to lateral epicondyle
Right	3.0	5.5	7.8
Left	3.0	5.5	7.6

## Discussion

In the cubital fossa, the median nerve lies medial to the brachial artery, deep to the bicipital aponeurosis and anterior to the brachialis muscle [2-3]. We found this nerve to lie an average of three centimeters lateral to the medial epicondyle. It is closely related to the brachial artery throughout its course in the arm [3]. It then enters the forearm between the heads of the pronator teres [3]. The median nerve innervates the palmar skin of the thumb, index and middle fingers and the lateral side of the ring finger and provides innervation to most of the flexor compartment of the forearm.

The radial nerve at the elbow lies in the deep groove between the brachialis medially and the brachioradialis and extensor carpi radialis longus, laterally [2-3]. It provides innervation to the extensors of the forearm and innervates the skin of the lower lateral arm, the posterior aspect of the forearm and the skin over the dorsum of the radial half of the ring, middle, and index fingers and the thumb [2]. We found the radial nerve to lie an average of 5.5 cm lateral to the medial epicondyle.

Our study shows that there was a difference in the distance from the medial epicondyle to the median and radial nerves between males and females. The median nerve is about 0.4 cm closer to the medial epicondyle in women compared to men and that the radial nerve is about 1.9 cm closer in women than men. This should be kept in mind when doing procedures at the elbow joint.

Injury to the median or radial nerve can have major implications including loss of extension, flexion, and sensation in the forearm and hand. A case report by Haapaniemi, et al. demonstrated complete transection of the median and radial nerves post arthroscopic debridement and capsular release in a right elbow [4]. Postoperatively, the patient complained of loss of sensory and motor function in the forearm and hand, clinical examination suggested the complete loss of function in the radial and median nerves [4].

Hackl, et al. examined twenty-two cadaveric upper extremities to analyze the location of the radial and median nerves in relation to bony landmarks in the ventral aspect of the elbow joint [1]. They concluded that the radial nerve can be found ventral to the central third of the capitulum in most cases, but Omid, et al. reported that the radial nerve courses medial to the capitulum [1, 5]. Hackl, et al. also found less variability in the median nerve, which was predominantly located along the medial quarter of the humeral epicondyle [1]. They found the radial and median nerves lie approximately 11 and 12 mm ventral to the capitulum and the trochlea, respectively [1]. Hackl, et al. concluded that the close proximity of the median and radial nerves to the ventral aspect of the elbow joint can increase the risk of nerve transection during arthroscopic anterior capsulectomy [1].

There have been several other studies on the relationship between arthroscopic sheath placement and nerve location at the elbow joint. Lynch, et al. found that the radial nerve is within seven mm and the median nerve within six mm of the arthroscopic sheath, using lateral and medial approaches respectively [6]. Marshall, et al. concluded that injury to the radial nerve is a complication of the anterolateral approach and injury to the median nerve a complication of anteromedial approach in elbow arthroscopy [7].

We should also mention certain techniques and maneuvers that can be used to change the location of the nerves running through the elbow joint. Studies by Miller, et al., Omid, et al., and Marshall, et al. have examined the different positions of the median and radial nerves with insufflation of the elbow joint and changing its position [5, 7-8]. 

## Conclusions

Easily identifiable landmarks are useful for localizing and avoiding regional neurovascular structures. The medial epicondyle can be readily assessed by the physician and thus serves as a good bony landmark. Our study measured the distance between this structure and the median and radial nerves in the cubital fossa. These measurements might be useful to those who wish to identify or avoid these nerves with various regional procedures such as venipuncture or surgery. 
